# Supplementary material for: Mutations in PCYT2 disrupt etherlipid biosynthesis and cause a complex hereditary spastic paraplegia
Source: Brain. 2019 Oct 22;142(11):3382–97. doi: 10.1093/brain/awz291 (PMC6821184; doi:10.1093/brain/awz291)
Supplement: awz291_Supplementary_Data [file awz291_supplementary_data.zip › awz291-Suppl_data/Supplementary_Data4.zip!/Supplementary information 4 - plasma lipidomics.zip!/Plasma lipidomics/Control vs Patient2/Statistical plots and data/Heatmap VIP-score bigger than 1.pdf]

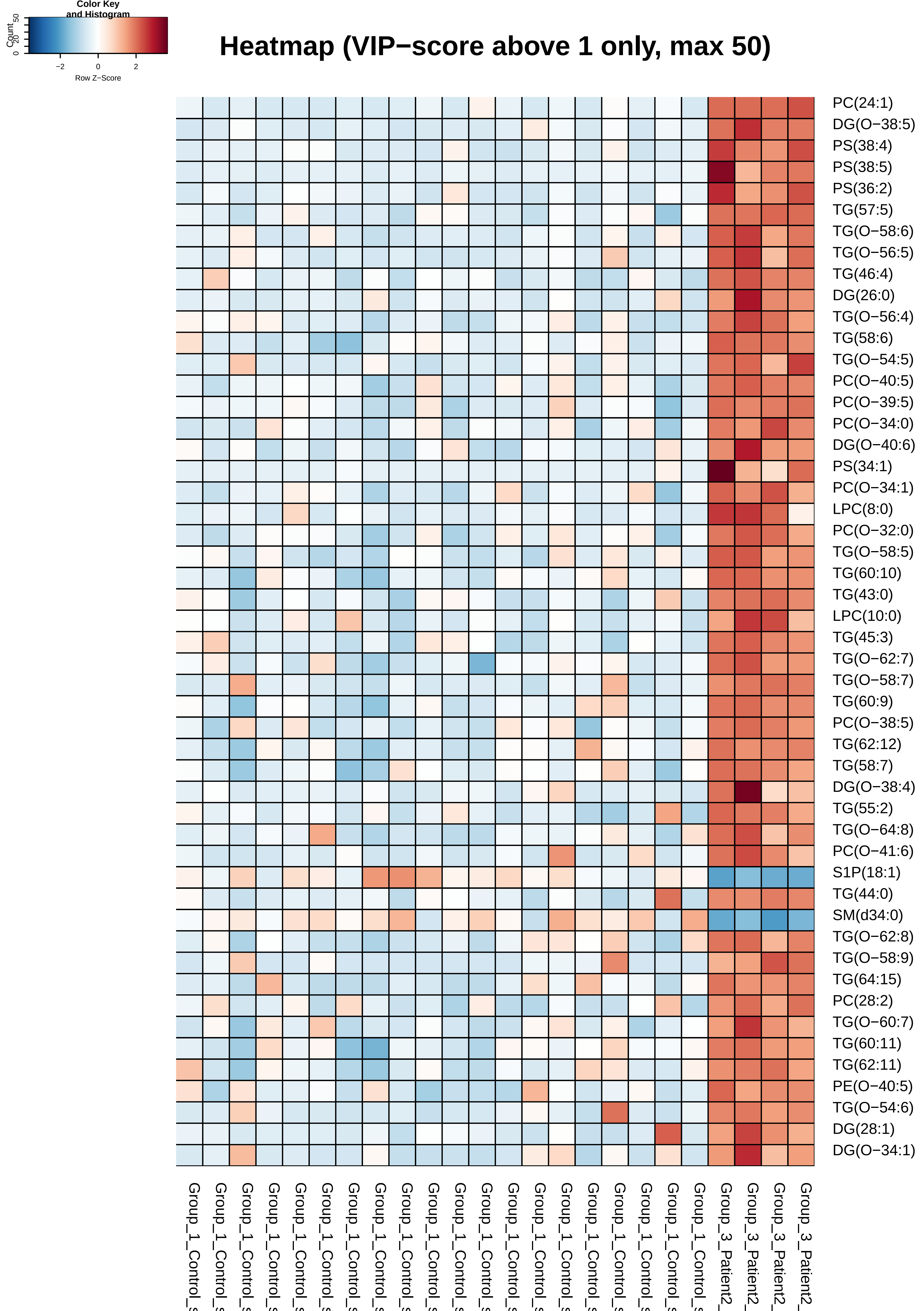

Color Key and Histogram
Count
0
20
50
−2
0
2
Row Z−Score
Heatmap (VIP−score above 1 only, max 50)
PC(24:1)
DG(O−38:5)
PS(38:4)
PS(38:5)
PS(36:2)
TG(57:5)
TG(O−58:6)
TG(O−56:5)
TG(46:4)
DG(26:0)
TG(O−56:4)
TG(58:6)
TG(O−54:5)
PC(O−40:5)
PC(O−39:5)
PC(O−34:0)
DG(O−40:6)
PS(34:1)
PC(O−34:1)
LPC(8:0)
PC(O−32:0)
TG(O−58:5)
TG(60:10)
TG(43:0)
LPC(10:0)
TG(45:3)
TG(O−62:7)
TG(O−58:7)
TG(60:9)
PC(O−38:5)
TG(62:12)
TG(58:7)
DG(O−38:4)
TG(55:2)
TG(O−64:8)
PC(O−41:6)
S1P(18:1)
TG(44:0)
SM(d34:0)
TG(O−62:8)
TG(O−58:9)
TG(64:15)
PC(28:2)
TG(O−60:7)
TG(60:11)
TG(62:11)
PE(O−40:5)
TG(O−54:6)
DG(28:1)
DG(O−34:1)
Group_1_Control_s
Group_1_Control_s
Group_1_Control_s
Group_1_Control_s
Group_1_Control_s
Group_1_Control_s
Group_1_Control_s
Group_1_Control_s
Group_1_Control_s
Group_1_Control_s
Group_1_Control_s
Group_1_Control_s
Group_1_Control_s
Group_1_Control_s
Group_1_Control_s
Group_1_Control_s
Group_1_Control_s
Group_1_Control_s
Group_1_Control_s
Group_1_Control_s
Group_3_Patient2_
Group_3_Patient2_
Group_3_Patient2_
Group_3_Patient2_
